# Motor modules account for active perception of force

**DOI:** 10.1038/s41598-019-45480-w

**Published:** 2019-06-20

**Authors:** Simone Toma, Marco Santello

**Affiliations:** 1 Laboratory of Neuromotor Physiology, Santa Lucia Foundation, Rome, 00179 Italy; 2 School of Biological and Health Systems Engineering, Arizona State University, Tempe, AZ 85287-9709 USA

**Keywords:** Human behaviour, Human behaviour, Statistics, Statistics

## Abstract

Despite longstanding evidence suggesting a relation between action and perception, the mechanisms underlying their integration are still unclear. It has been proposed that to simplify the sensorimotor integration processes underlying active perception, the central nervous system (CNS) selects patterns of movements aimed at maximizing sampling of task-related sensory input. While previous studies investigated the action-perception loop focusing on the role of higher-level features of motor behavior (e.g., kinematic invariants, effort), the present study explored and quantified the contribution of lower-level organization of motor control. We tested the hypothesis that the coordinated recruitment of group of muscles (i.e., motor modules) engaged to counteract an external force contributes to participants’ perception of the same force. We found that: 1) a model describing the modulation of a subset of motor modules involved in the motor task accounted for about 70% of participants’ perceptual variance; 2) an alternative model, incompatible with the motor modules hypothesis, accounted for significantly lower variance of participants’ detection performance. Our results provide empirical evidence of the potential role played by muscle activation patterns in active perception of force. They also suggest that a modular organization of motor control may mediate not only coordination of multiple muscles, but also perceptual inference.

## Introduction

While judging the property of an object (e.g., weight, shape or texture) through active interaction, people select specific movements in accordance to the features to be judged^1^. Accordingly, it has been proposed that in active perception, detection and discrimination of stimulus features depend on the motor-sensory associations arising from the exploratory movements^2^. Following this hypothesis, active perception would rely on the coupling between movements and patterns of movement-generated sensory inflow^3^. This framework was first tested by a series of studies revealing that features of the motor response can influence perceptual judgment^4,5^. More recent investigations have supported this view by showing that action-related characteristics of the task – such as effort or biomechanical constraints - can shape the interpretation of the sensory signals underling perceptual decision making^6–8^.

While these previous studies focused their attention on higher level features of motor behavior (e.g., kinematic invariants, effort), the present study investigated whether and to what extent lower-level organization of motor control contributes to active perception. Specifically, we tested the hypothesis that participants’ detection of an external force depends on the coordinated recruitment of groups of muscles engaged to counteract the same force.

**Figure 1:**
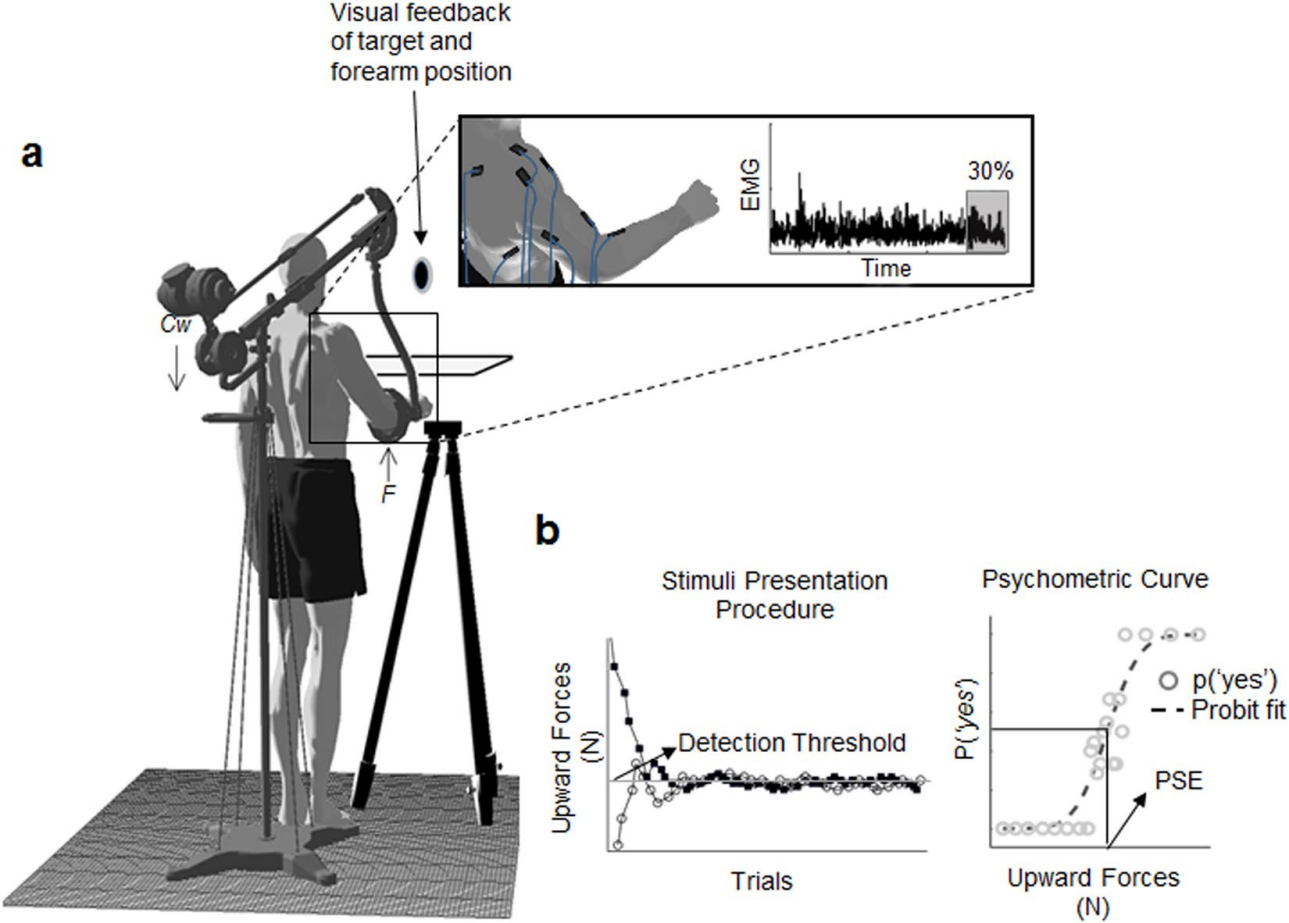
Apparatus. (**a**) Track-hold (TH) device and participants’ arm posture at the moment of force application, i.e., cursor aligned with the target sphere and arm posture not exceeding 5.0° from the initial arm configuration (see *apparatus* section). *Cw*: load applied on TH balancer. *F*: resultant upward force applied on participants’ arm. The inset shows the eight electrodes applied on forearm and upper arm muscles: brachio-radialis, biceps brachii, long head, triceps brachii, long head; trapezius upper; trapezius middle; latissimus dorsi; deltoid, anterior and deltoid, posterior. The EMG dataset considered for the analysis was composed of the last 30% of each trial total duration. **b**) *left:* Double staircase force presentation procedure. White and black circles and markers represents the ascending and descending staircase, respectively. Horizontal line indicate the force intensity where the two stairs converged. *Right:* probabilities of detection (circles) and probit curve fit. PSE was calculated as the force intensity eliciting 50% of probabilities of answer yes.

We asked participants to judge the presence of an upward force (yes/no answer) applied on their forearm while maintaining the arm in a quasi-isometric posture against the force stimulus (Fig. 1a). We derived the psychometric curves describing participants’ probabilities of detection as a function of the external force (Fig. 1b). Using the EMG signals recorded from eight shoulder and elbow muscles (Fig. 1a inset), we developed two probabilistic models describing the modulation of the muscle activity involved in the task. The first model was compatible with the theory of motor primitives^9^ positing that force production during natural movements is generated by the combination of muscle synergies, i.e., motor modules^10,11^. We refer to this model as *muscle synergy model* (MSM). The second model, incompatible with the muscle synergy hypothesis, was generated based on the assumption that concurrent activity of multiple muscles during force production relies on flexible recruitment of individual muscles^12,13^. We refer to this model as *most contributive model* (MCM). We tested our hypothesis that motor modules can account for active perception of force by assessing whether: 1) the probability of force detection is better accounted for by a muscle-model that takes into account the modulation of muscle synergies rather than a linear combination of activity of individual muscles; 2) the model accounting for the highest amount of perceptual variance requires only a subset of the muscle synergies recruited in the motor task; and 3) the two muscle models differ in terms of the patterns of muscle activity accounting for the highest amount of perceptual response variance.

## Results

**Figure 2:**
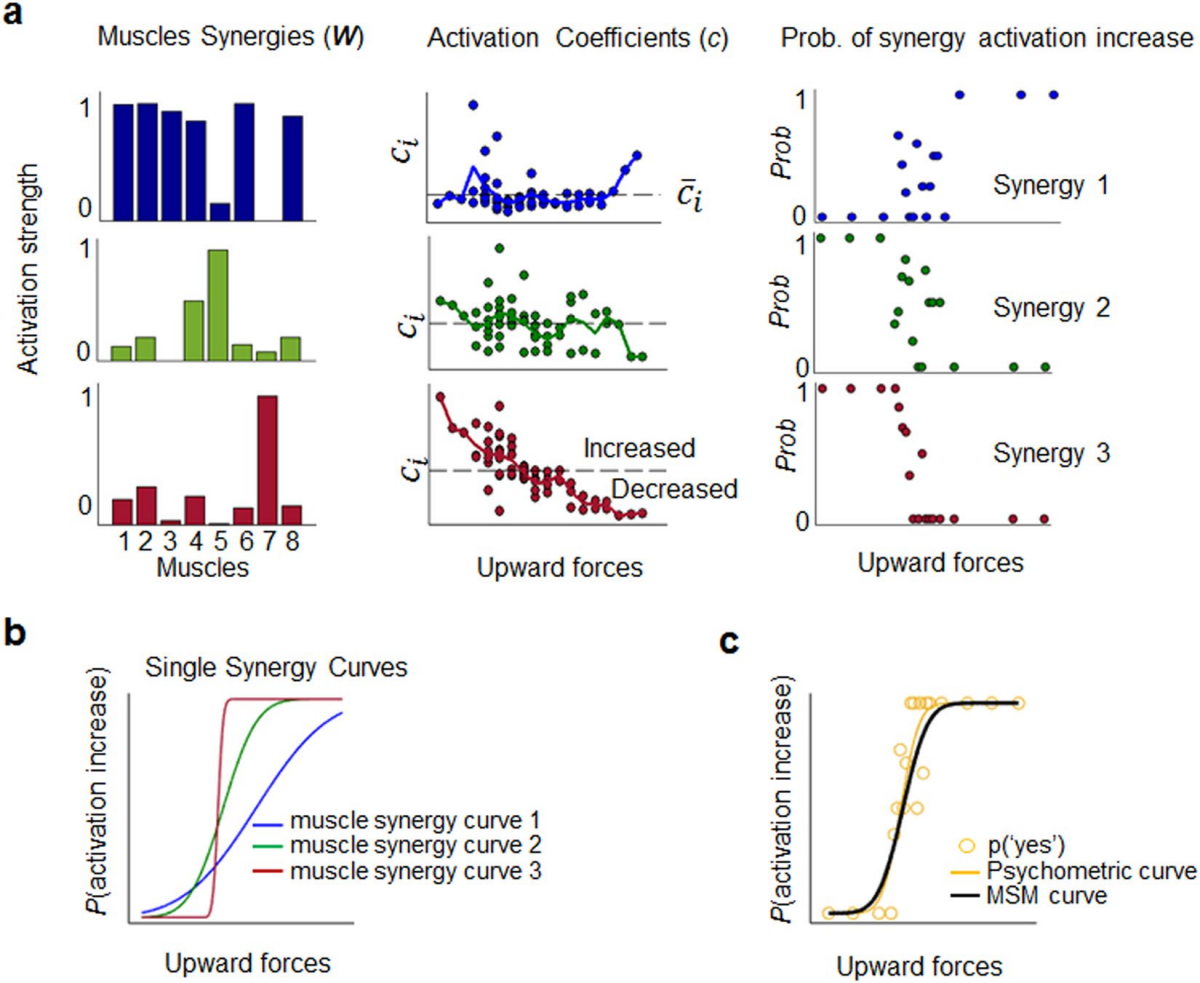
Synergy extraction and force-related synergy identification. (**a**) Least number of muscle synergies (W) and associated activation coefficients *c* that account for 90% of the EMG total variance (i.e., task related synergies) of a representative participant (i.e., subj 1). Left plots: Activation strength of the muscle synergies extracted from eight arm muscles: brachioradialis (1), biceps brachii (2), triceps brachii (3), trapezius middle (4) and upper (5), latissimus dorsi (6), deltoid anterior (7) and posterior (8). Middle plots: each dot describes the amount of activation *c* of the synergy *i* as function of the external upward force. Multiple dots associated to the same upward force represents different trials with the same force intensity. The horizontal dashed line indicates the mean value of the distribution of the activation coefficient across all trials for each synergy ($\overline{{C}_{i}}$). At each trial *t*, each synergy activation was classified as increased when ${C}_{it} > \,\overline{{C}_{i}}$, or decreased when ${C}_{it} < \,\overline{{C}_{i}}$. Right plots: Dots represent the across-trials probabilities of each external force to elicit an activation increase of the synergy *i*. (**b**) Individual synergy curves (GLMM output) relating the probabilities of each synergy activation increase/decrease as a function of force stimuli. For each curve, slope values quantify the amount of modulation of the muscle synergy as function of force stimuli. **c**) GLMM overall predicted probabilities of activation increase (black curve) of the subset of muscle synergies showing statistical significant modulation with respect to force stimuli. Overall muscle-synergy -i.e., MSM - curve was obtained through an iterative procedure identifying and excluding, at each iteration, the synergy modules not significantly related or weakly encoding force stimuli (see *Force-related synergy identification and MSM curve*, Methods). In this example, the overall muscle-synergy curve is drawn by the GLMM analysis combining synergy 2 and 3 probabilities of activation increase, synergy 1 being excluded (see details in *Force-related synergy identification and MSM curve*, Methods). Yellow circles and curve denote probabilities of a *‘yes’* answer and the psychometric curve, respectively, describing participants’ perceptual behavior as function of the applied force (GLM probit fitting).

We recorded EMG signals from eight shoulder and elbow muscles (Fig. 1a inset) during an active force detection task to test the hypothesis that the modulation of a subset of muscle activations patterns (i.e., synergies), involved in counteracting the external force, can explain a substantial portion of participants’ perceptual response variance. Specifically, from the group of synergies recruited during the isometric task, i.e., *task-related synergies* (Fig. 2a,b), we extracted the subset of muscle modules whose activation significantly correlated with the magnitude of force stimuli, i.e., *force-related synergies*. We then assessed the similarity between a curve describing the across-trials modulation of the force-related synergy (Muscle Synergy Model, MSM, curve) and the psychometric curve describing the probability of force detection (Fig. 2c). We cross-validated the hypothesis that motor modules contribute to force perception by estimating the amount of perceptual variance explained by an alternative model incompatible with the MSM hypothesis, i.e., the Most Contributive Model (MCM). While the MSM is based on patterns of muscle activity associated with the motor task, the MCM describes force detection associated with activity of individual muscles weighted with respect to their modulation.

### Force detection thresholds

For each participant, the psychophysical procedure employed during the experiment ensured the identification of a force detection threshold, i.e., Point of Subjective Equality, PSE). Specifically, the adaptive method used for the presentation of the force stimuli allows estimation of a force intensity above and below which participants increased the probability of reporting the presence or absence of the upward force, respectively (Fig. 1b). Participants’ perceptual performance in detecting the presence of the upward force was assessed through the probit analysis fitting the probabilities of a ‘yes’ answer as a function of force stimuli (Fig. 1b). The PSE and slope (sensitivity to force changes), estimated by 1000-sample bootstrapping, revealed inter-subject differences in both participants’ PSE and slope. Across participants, PSE ± SD ranged between 0.1 ± 0.1 N and 18.9 ± 0.7 N (Fig. S1 and Table S1 in Supplementary Material). These results indicate that while some participants were able to modulate their answers – i.e., detection or no detection – within a narrow range of forces (slope approaching 1), other participants required a wider step change of force stimulus intensity to reverse their judgment. Importantly, despite idiosyncratic sensitivities to changes in force stimuli, the intercepts and slopes obtained by probit analysis were statistically significant for all participants (Table S1 in Supplementary Material). Similarly, deviance tests of the psychometric curves accounting for participants’ probability of force detection revealed deviance values that were not significantly different than zero, hence supporting the goodness of our fits. Together, these results confirmed that the psychometric curves provided a reliable description of the participants’ perceptual responses as a function of force stimuli.

### Task-related muscle synergies

**Figure 3:**
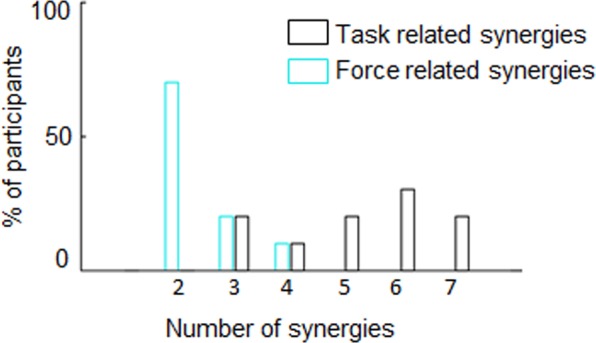
Number of synergies composing the task- and force-related subset. Across-participants (n = 10) frequency distribution of the number of muscle synergies composing the task-related subset (black) and force-related subset (cyan). The task-related subset is composed of the least number of muscle modules reconstructing over 90% of the whole EMG dataset variance. The force-related subset is composed by the smallest set of muscle synergies modules significantly related to the external force (GLMM analysis).

The activity of eight arm muscles observed while counteracting the external force revealed a small number of activity modules, i.e., task related synergies, accounting for over 90% of the overall EMG data variance (Fig. 2a). We found, on average ( ± SD), 5.2 ± 1.5 task-related synergie*s* (Fig. 3, black empty bars). For all participants, these synergies explained more than 75% of the variance of activity of individual muscles and more than 90% of variance of the overall muscle activity variance (Table 1; see S1.1 and Fig. S2 in Supplementary Material). The comparison of variance accounted for (VAF) by the real EMG dataset and structure-less data (see *Muscle synergy extraction*, Methods section) supports the reliability of our analysis. Specifically, for each participant, the extracted global VAF from random data reconstruction was always significantly lower than the global VAF obtained from task-related synergies. Across participants, random and actual VAF median ± SE were respectively 64 ± 8% and 97 ± 1% (Wilcoxon two-sided paired test: n = 10, *z* = 2.6, *p* = 0.003, *ƞ*^2^ = 0.82; see Table 1 for individual subjects’ results).

**Table 1:** Synergy extraction parameters. Minimum number of muscle synergies able to satisfy both local (VAF > 75%) and global reconstruction criteria (VAF > 90%). VAF values are reported as median and confidence intervals of variance accounted for. Z-statistics (right-sided Wilcoxon signed rank test) tested whether global VAF extracted from actual data was higher than VAF obtained from random data. ** for *p*-values < 0.01.

	*Synergies*	*globalVAF (act)*	*Z-statistic*
*Subj. 1*	3	91% (91; 92)	12.26**
*Subj. 2*	5	96% (95; 96)	12.26**
*Subj. 3*	5	94% (93; 95)	12.26**
*Subj. 4*	4	98% (97; 98)	12.26**
*Subj. 5*	7	99% (98; 99)	11.29**
*Subj. 6*	6	94% (93; 96)	12.26**
*Subj. 7*	3	92% (91; 93)	12.26**
*Subj. 8*	6	98% (97; 99)	12.01**
*Subj. 9*	7	99% (98; 99)	9.62**
*Subj. 10*	6	98% (96; 99)	11.77**

### Force-related synergies

Analysis of the probability of each muscle synergy to increase its activity (activation coefficient; see Methods) as function of the force stimulus allowed us to identify the minimum number of muscle modules encoding for external forces (see Fig. 2a and *Force-related synergies identification and MSM curve*, Methods). Our results show that participants’ muscle activity during the isometric task was mainly characterized by two types of muscle modules: one engaged in counteracting the external force (force-related synergy; synergy 2 and 3, Fig. 2a), and the other recruited to maintain arm posture (postural synergies), hence showing weak modulation as a function of changes in the external force and concurrent activation of flexor and extensor muscles (synergy 1, Fig. 2a; see Fig. S3 in Supplementary Material for individual subjects’ data). In contrast, force-related synergies were characterized by stronger modulation of activation coefficients and activation of flexor muscles as a function of force stimulus magnitude (synergy 2 and 3, Fig. 2a,b). For each participant, the curve describing the probability of the modulation of all synergies as function of force exhibited statistically significant slope parameters and goodness of fit (p < 0.01, χ² statistics; Fig. 2c. These results indicate that the MSM could reliably describe the modulation of both individual force-related synergies and all force-related synergies (color and black curves, Figs. 2b,c, respectively; see *Psychometric and muscle synergy curve*, Methods, and Fig. S3, Supplementary Material). Importantly, the above-described differences in sensitivity to force stimuli between force-related and postural synergies were found in all participants (see Fig. S3, Supplementary Material). We found, on average, 2.4 ( ± 0.7) force-related synergies (Fig. 3).

### Comparison between MSM and psychometric curve

To test the hypothesis that muscle modules involved in counteracting an external force can account for participants’ force detection, we estimated the similarity (deviance ratio and delta parameters; see Methods) between the probability curves of participants’ force stimulus detection (psychometric curve) and modulation of force-related synergy activation (MSM curve), both as function of force stimuli.

**Figure 4:**
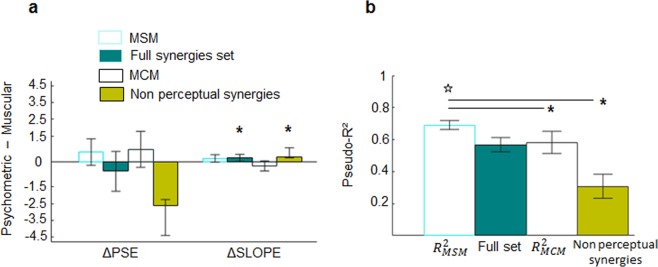
Comparison of muscle models. (**a**) ΔPSE and ΔSLOPE medians ± SE (n = 10) extracted from the comparison between the parameters describing the psychometric curve and the curve predicted by MSM (cyan empty bar), the full set of synergies (green), MCM (black empty bars). and the subset composed of non-perceptual synergies (light green). Distribution of ΔSlope and ΔPSE were submitted to one sample Wilcoxon one-tailed test to assess whether medians were significantly different from 0 (p < 0.05). (**b**) Efron pseudo-R² medians ± SE (n = 10) describe the amount of perceptual variance, i.e., P(*yes*), accounted for by the curves derived from MSM, MCM, full set of synergies and non-perceptual synergy subset (color code as in **a**). Statistically significant differences across pseudo-R² values were tested through a non-parametric Friedman ANOVA. Post-hoc tests were conducted by applying Bonferroni correction, * indicating p < 0.008. Star denotes the one-tailed Wilcoxon non-parametric test assessing models’ pseudo-R² medians significantly higher than 0.6.

For most participants (8/10), the curve describing the modulation of force-related synergies was statistically indistinguishable from the psychometric curve (deviance ratio, Table 2; see Fig. S4 in Supplementary material for individual subjects’ data). We found that the overall MSMs’ *Δ*_*pse*_ and *Δ*_*slope*_ median ± SE values (n = 10; 0.55 ± 0.42 and 0.18 ± 0.12, respectively) were not significantly different than 0 (p = 0.37 and 0.08, respectively; Fig. 4a). A two-tailed Wilcoxon signed rank test of pseudo-*R*^2^ values revealed MSM to account for more than 60% of the perceptual variance (0.69 ± 0.03; p = 0.004, *ƞ*^2^ = 0.81; Table 2 and Fig. 4b). We note that for participants 4 and 8, the MSM curve was significantly different from the psychometric curves (deviance ratio 11.08 and 9.56 for participants 4 and 8, respectively, *p* < 0.01; Table 2). This indicates that a statistically significant relation between the modulation of force-related synergies and force stimuli does not guarantee, by itself, a correlation between muscle activity and force perception.

**Table 2:** Perceptual synergies. The deviance ratio measures the relation between the deviances obtained by fitting the psychometric and the synergy curve on the probabilities of the *‘yes’* answer. Deviance ratio not exceeding χ² critical value with 2 degrees of freedom (** for p < 0.01) indicates that synergy curve describes perceptual behavior no worse than the psychometric function.

	Deviance ratio	*Δ*_*slp*_	*Δ*_*pse*_	${{\boldsymbol{R}}}_{{\boldsymbol{MSM}}}^{{\bf{2}}}$	${{\boldsymbol{R}}}_{{\boldsymbol{MCM}}}^{{\bf{2}}}$
*Subj. 1*	1.21	0.01	−0.74	0.85	0.70**
*Subj. 2*	1.15	−0.1	1.27	0.64	0.13**
*Subj. 3*	1.01	−0.04	−2.2	0.69	0.47**
*Subj. 4*	11.08**	0.67	−0.79	0.80	0.76**
*Subj. 5*	3.09	0.40	−1.6	0.84	0.79**
*Subj. 6*	3.76	0.25	2.33	0.57	0.50**
*Subj. 7*	1.79	−0.06	−1.33	0.70	0.54**
*Subj. 8*	9.56**	1.04	−0.31	0.69**	0.71
*Subj. 9*	1.92	0.47	0.86	0.69	0.62**
*Subj. 10*	0.98	−0.15	−0.37	0.65	0.25**

*Δ*_*slp*_ and *Δ*_*pse*_ were obtained by subtracting synergy curve parameters from those describing the psychometric function. Efron pseudo-${R}_{MSM}^{2}$ values quantify perceptual answers variance accounted for the synergy model. Efron pseudo-${R}_{MCM}^{2}$ represents the perceptual answers variance accounted for the most contributive model. ** denotes two-sided Wilcoxon signed rank test (p < 0.01) assessing whether the median of the ${R}_{MCM}^{2}$ distribution is significantly different than those associated with ${R}_{MSM}^{2}$ value.

### MCM curve and perceptual variance

**Figure 5:**
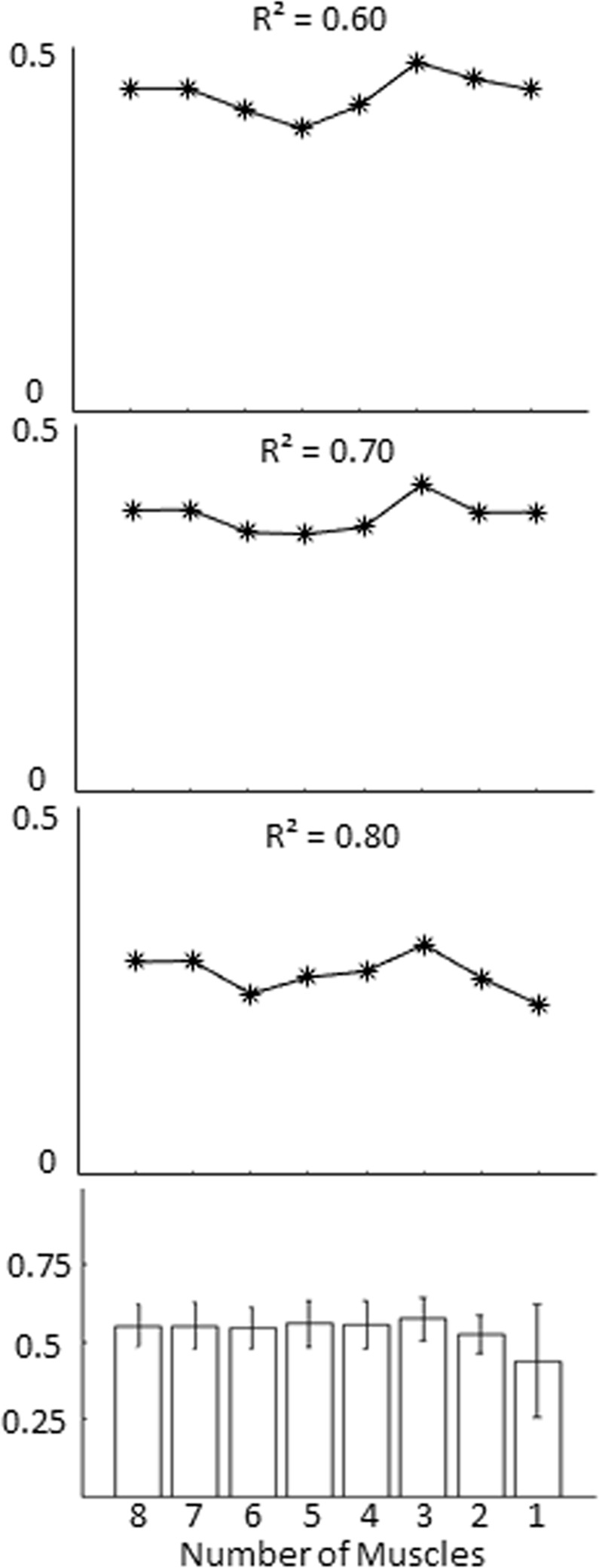
Best MCM curve. From top to bottom, the first three plots show the likelihood (*L*(R² | Number of Muscles) of the MCM curve’s ability to provide 60% to 80% of explained perceptual variance (pseudo-R²) as a function of the number of muscles (1 to 8). The bottom plot shows the median ± SE of across-participants pseudo-*R²*. The likelihood of the variance accounted for by each MCM was obtained through an iterative multiple regression with a backward elimination procedure where, at each iteration, the least contributive muscle was excluded from the model (for details see *The most contributive model*, Methods). This analysis shows that the MCM composed of three muscles is always the most likely to explain approximately 60% (or higher) total perceptual variance.

The Most Contributive Model (MCM) curves describe the modulation of the weighted sum of activity of a variable number of muscles (see Methods). The hypothesized role of MCM on force perception in our study is based on the proposition that the way in which muscles are recruited may be instrumental in reducing signal-dependent noise^14^ (see Supplementary Material S1.3 for details). The comparison between MCM and psychometric curves revealed that the MCM curve describing the weighted sum of activity of three muscles always provided the highest likelihood to explain at least 60% of perceptual variance (Fig. 5, top three plots). Moreover, all *R²* values associated with the seven MCM nested curves – describing the overall activity obtained by combining 8 to 2 muscles – were not significantly different than 0.6 (two-tailed Wilcoxon signed rank test, *p* > 0.05; Fig. 5, bottom plot; see Table S2 in Supplementary Material for details). Specifically, median *R*^²^ ± SE ranged from 0.55 ± 0.02 (nested model composed of 8 muscles) to 0.58 ± 0.01 (nested model composed of 3 muscles). These results suggest that a model developed from the linear combination of the activity of only three muscles, all highly modulated during the isometric task, can provide a description of participants’ force detection over chance level.

### MSM outperforms MCM perceptual description

We have shown that both a weighted sum of individual muscle activity and a synergy-based description of muscle activity involved in an isometric task can explain a large portion of perceptual variance. To test our hypothesis that motor modules play a significant role in active perception of force, we compared the performance of MSM with MCM by testing for differences of their median pseudo-*R*^2^ and curve parameters obtained from all participants. Median *Δ*_*pse*_ and *Δ*_*slope*_ ( ± SE) values were 0.72 ± 0.58 and −0.26 ± 0.16, respectively, for the MCM curve and 0.55 ± 0.43 and 0.18 ± 0.12 for the MSM curve (Table 2 and Fig. 4a). *Δ*_*pse*_ and *Δ*_*slope*_ calculated from the psychometric and MCM curve were higher than the *Δ* values associated with the MSM curve (see Fig. S4 in Supplementary Material for individual curve comparisons). Moreover, both the Δ parameters extracted from the comparison between the psychometric and the MSM curve did not statistically differ from zero (p = 0.37 and 0.08 for *Δ*_*pse*_ and *Δ*_*slope*_, respectively). Furthermore, the MCM curve accounted for less perceptual variance than the MSM curve for 9 out of 10 participants (Fig. 4b; see Table 2 for statistical analysis of individual subjects). Unlike the MSM, the pseudo-*R*^2^ associated with the MCM curve was not significantly different than 0.6 (0.58 ± 0.08; p = 0.84). Friedman ANOVA performed on the across-participants pseudo-*R*^2^ distributions of MCM, MSM and two different synergy curves (see *Cross-validation of the MSM hypothesis* below) describing p(yes) revealed significant differences in the amount of perceptual variance explained across models (χ²(3) = 17.76; p = 0.0005). Post hoc analysis with Wilcoxon signed-rank tests (Bonferroni p-value corrections, *p* = 0.05/6 = 0.008) revealed MCM pseudo-*R*^2^ was significantly lower than MSM pseudo-*R*^2^ (p = 0.0041; *ƞ*^2^ = 0.86; Fig. 4b). Together, these results suggest that the muscle-synergy based model outperformed the description of force detection provided by the linear model by ~11% of explained variance.

### Cross-validation of the MSM hypothesis

We considered the possibility that the advantage of the MSM model in describing perception might have depended on its greater ability to reconstruct muscle activity. Specifically, the advantage of the MSM might be attributable to the fact that the muscle synergy decomposition analysis captures more task-dependent EMG variability, hence force detection, than the weighted linear combination approach. If this were the case, the MSM curve drawn from all synergies (full set) would improve the similarity with the psychometric curve, as this would lead to a complete reconstruction of the EMG signals (VAF = 100%). However, we found that using the full set of muscle modules to create MSM curves does not enhance force detection, even though it improves EMG reconstruction (Fig. 4a,b). Specifically, the full set of synergies provided a *Δ*_*slope*_ statistically different than zero (0.21 ± 0.12, p = 0.006) and a pseudo-*R*^2^ (0.57 ± 0.05) not significantly different than 0.6 (p = 0.37). This suggests that MSM’s better performance than MCM in accounting for force detection was not caused by its ability to reconstruct EMG activity (see *Methodological considerations* in Discussion).

We also considered the possibility that force-related synergies might have favored the MSM by virtue of the relation between EMG and force stimulus magnitude. If this were the case, one might expect any of the force-related muscle synergies recruited during the motor task to be similar with the psychometric curve, regardless of the extent of their modulation with external force. However, we found that the force-related synergies that were weakly or not modulated by the force stimuli (i.e., non-perceptual synergies; see *Comparison between muscle-model curves and perceptual performance* in Methods) account for less variance than the force-related synergies used for the MSM. Specifically, *Δ*_*slope*_ was significantly different than zero (0.25 ± 0.10, p = 0.002; Fig. 4a) and a pseudo-*R*^2^ statistically lower than 0.6 (0.31 ± 0.07; one-sided Wilcoxon signed rank test: p < 0.001) (Fig. 4b). Friedman ANOVA post-hoc paired comparisons performed on the pseudo-*R*^2^ distributions of the MSM, and both the full and non-perceptual synergy models, revealed the synergy model accounted for significantly higher perceptual variance than the non-perceptual model (p = 0.002, Fig. 4b) and full model (p = 0.02, Fig. 4). (see *Methodological considerations* in Discussion).

**Figure 6:**
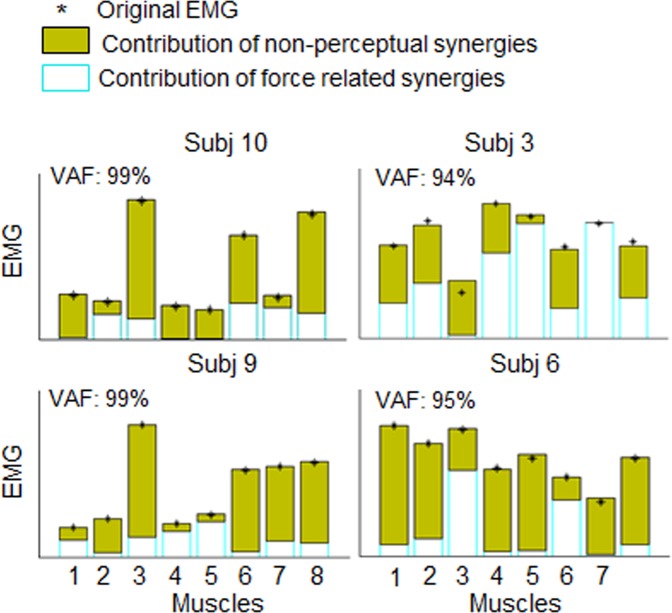
Contribution of synergy subsets to EMG dataset reconstruction. Perceptual (cyan empty bars) and non-perceptual (filled green bars) synergy subset contribution to the reconstruction of the original EMG activity (VAF) of each muscle calculated (4 representative participants; for data from the remaining participants see Fig. S6, Supplementary Material). Asterisks denote original EMG from each muscle. VAF values refer to the global variance accounted for by the group of task-related muscle synergy, namely when perceptual and non-perceptual synergies subsets were merged for EMG reconstruction. Muscles numbering is the same as in Fig. 2.

Finally, we tested whether the superior performance of MSM was due to the greater ability of force-related synergies than non-perceptual synergies to capture more variance of muscle activity involved in the isometric force production task. If so, MSM should account for greater perceptual variance (pseudo-*R*^2^) and EMG dataset reconstruction (VAF) than a subset of non-perceptual synergies. The contributions of each subset of synergies in reconstructing the overall muscle activity is shown for four representative subjects in Fig. 6 (data from all participants are shown in Fig. S6, Supplementary Material). Despite its better description of force perception, MSM subset of muscle modules contributes equally or less than the non-perceptual synergies to the reconstruction of the original EMG dataset. This finding indicates that, although both force- and non force-related synergies were recruited during our isometric task, only the former subset influenced force detection.

### Coefficient of shared muscles

**Figure 7:**
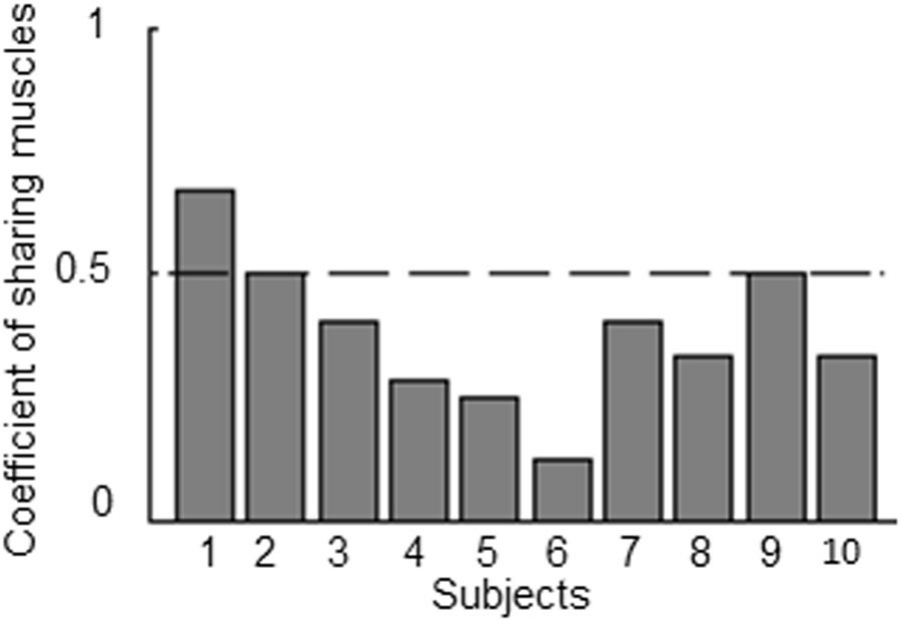
Coefficient of Shared muscles. Coefficient of shared muscles denotes the proportion of the muscles with activation strength higher than 0.5 in MSM and that are also in the MCM best model. Lower coefficient values indicate that MSM accounts for perceptual variance by means of different muscles than those belonging to the MCM.

Our last analysis focused on assessing whether the muscles that were active the most within the MSM synergy subset were also the muscles included in the best MCM, i.e., explaining the highest about of perceptual variance with the smallest number of muscle (see *The Most Contributive Model* in Methods). Specifically, if MSM and MCM shared the same muscles, the muscle modules identified by the former approach would be characterized by high activation strength of the same three muscles composing the MCM. This would result in a coefficient of shared muscles close to 1 (see *Muscle model composition and synergies contribution*, Methods). Figure 7 shows the proportion of muscles shared by both MCM and MSM for each participant. We found that only one participant (i.e., subject 1) exhibited a coefficient of shared muscles higher than 0.5. Across participants, the coefficient of shared muscles ranged from 0.12 and 0.67 (median ± SE: 0.36 ± 0.05). Overall, about 60% of the muscles underlying the MSM curves were different from those included in the MCM curves.

## Discussion

The present study provides evidence that a subset of motor modules describing patterns of muscle activation involved in an isometric motor task may underlie active perception of force. Specifically, we showed that a model based on modulation of a subset of muscle activity patterns (i.e., muscle synergies), MSM, explained approximately 70% of participants’ perceptual variance of force stimulus detection (Fig. 4b). We also demonstrated that a model incompatible with the muscle synergies hypothesis that assumes a linear combination of individual muscles activity, i.e., MCM, described perceptual behavior significantly worse than the MSM (Fig. 4b, black empty bar). Remarkably, our data revealed that the MSM’s ability to explain perceptual variance did not depend on the ability of the model to reconstruct a larger portion of the original EMG dataset (Fig. 6). Moreover, as hypothesized MCM and MSM described perception by means of different muscle combinations (Fig. 7). Below we discuss our findings, the extent to which a modular organization of muscle activity may contribute to active perception of force, methodological considerations and open questions.

### Multiple muscle activity and force perception

Our findings suggest that the greater ability of MSM relative to MCM in accounting for force perception stems from significant differences in how they capture the coordination of muscle activity involved in performing our isometric force production task. Thus, the present results support our initial hypothesis that the model accounting for the highest amount of perceptual variance was related to the modulation of only a subset of the muscle synergies recruited in the motor task. Importantly, perceptual variance accounted for by a model consisting exclusively of non-perceptual synergies was significantly reduced relative to the MSM (Fig. 4b). Similarly, a model composed of all muscle modules (i.e., full set of synergies) did not increase the similarity between the synergy and psychometric curves (Fig. 4a-b), even though it improved EMG reconstruction.

Our findings are in agreement with previous work suggesting that muscle synergies may facilitate the coordination of activity of multiple muscles and interpretation of muscle afferent signals^15,16^ underlying force perception. Gandevia and colleagues speculated that when multiple synergistic muscles are activated through muscle reflexes, perception of muscular effort is mediated by efferent and afferent signals associated to the whole group of muscles, rather than individual muscles^15,17^. These authors argued that, while muscle reflexes facilitate muscle coordination, they may also reduce the CNS ability to distinguish among different motor drives directed to individual muscles^17^. They further proposed that estimation of force is likely to arise from the combination of descending motor commands and modulation of reflex responses at motoneuronal and pre-motoneuronal levels^15^. The present results are consistent with this proposition by showing that the analysis of coordinated pattern of muscles activity provided by MSM explains a larger portion of perceptual variance than an approach, such as MCM, that is based on individual muscles whose contribution is weighted according to modulation of their activity. The assumption of a weighted sum of individual muscle activity that underlies the MCM is consistent with the hypothesis that sense of force would depend on the relation between descending motor commands and the features of the individual muscles group mainly involved in the task^18–20^, e.g., muscle length, muscle origin and insertion. Accordingly, previous work has suggested that the perception of force and weight is relative rather than absolute^21^. Our results are compatible with this proposition, as they suggest that the relation between perception and activity of multiple muscles provided by the MCM’s is likely to be found regardless of whether the motor task involves single or pairs of functionally-related muscles (see S1.3, Supplementary Material for further discussion).

To date, the only empirical evidence supporting a relation between synergistic muscle activity and force perception has been provided by studies of individuals affected by hemiparesis^22,23^. These studies have shown a relation between abnormal synergistic muscle activation caused by hemiparesis and bias in force perception. Thus, to the best of our knowledge, the present study is the first attempt to support the relation between muscle synergies and active perception of force through a detailed analysis of the muscle activity in healthy participants.

### Muscle modules in active perception

The observation that a subset of muscle synergies can account for active perception of force suggests the involvement of central mechanisms that combine task-related sensorimotor signals that are most relevant for a perceptual decision. Thus, we propose that during active perception, motor modules not only simplify motor control in terms of biomechanical demands of the task^24^ but also contribute to the extraction of task-relevant information. Such information consists of a subset of muscle modules encoding external force changes. This interpretation is supported by evidence suggesting that muscle synergies may facilitate the establishment and retention of recurring sensorimotor mappings across different motor tasks^25–27^. Our results support and extend the proposition that the transformation of sensorimotor signals into perceptual decisions is influenced by the strategy employed by the CNS to deal with effort^6^, costs^28^ and, as shown here, a redundant musculoskeletal system.

Based on the evidence provided by the present study, we speculate that perception associated with activation of multiple muscles may arise not just from sensory inputs conveyed by primary afferents or direct interpretation of the descending command^29,30^, but rather from detecting state changes in the circuitry of the spinal cord and integrating multimodal inputs to sensory and motor cortex. This speculation is consistent with recent neurophysiological studies proposing that hand synergies emerging during grasping are encoded by bimodal neurons integrating tactile with proprioceptive inputs in the somatosensory cortex^31^. Similarly, the presence of multimodal neurons responding to both efferent and afferent signals in the pre-motor, motor and parietal cortex^32,33^, suggests a correspondence among mirror neuron mechanisms, muscle synergies^34^ and active perception^3^. Within this network, the cerebellum is likely to contribute to active perception through the modulation of the fusimotor system and its projections to parietal cortex^35^. It has been proposed that one of the roles played by the spinal premotor interneurons is to facilitate muscle co-activations and integration of inputs from peripheral afferents with descending motor command^36^. Therefore, active perception may also depend on sensorimotor integration occurring in the spinal cord by means of muscle fields encoded in premotor interneurons^36,37^. Our results are also consistent with previous work proposing modularity as a CNS strategy to interpret synergy-related afferent information^38–40^. This framework is supported by recent studies proposing that premotor interneurons in the spinal cord do not simply “relay” neural activity from the motor cortex to motor units, but rather they integrate dynamic and static components of the motor command^36^.

In agreement with recent compelling evidence suggesting that the motor system can influence how sensorimotor signals are transformed into decision variables^8^, we interpret our findings as evidence that in active perception, patterns of activity of multiple muscles organized in muscle modules may simplify the sampling of relevant motor-sensory inputs^39,40^ driving a perceptual decision.

### Methodological considerations

It may be argued that the observed relation between muscle synergies and force perception is a direct and obligatory consequence of the task dynamics relating modulation of muscle activity to the changes in external forces. This proposition is supported by the observation that muscle synergies encode force magnitude, where synergy activation coefficients correlate with increases in force stimuli^41^. Nevertheless, in a previous report^42^ we showed no univocal relation between the applied force and upper limb muscle activity, nor between the applied force and the modulation of perceptual responses. Consistent with our previous work, the present results demonstrate that the muscle synergies subset that best accounted for perception did not account for a higher portion of overall muscle modulation, i.e., EMG reconstruction (Fig. 6 and S6 in Supplementary Material). Moreover, despite the high accuracy of EMG reconstruction provided by the task-related muscle synergies subset, two participants out of ten did not exhibit a significant relation between the modulation of synergy activation and the trend of their perceptual responses. Together, these findings indicate that the accurate description of the muscular activity involved in the motor task is not sufficient to account for the observed perceptual variance.

Another methodological consideration is whether participants might have made an implicit decision about the upcoming external force which, in turn, would have influenced the pattern of muscle activity we observed. However, our results do not favor this possibility. If participants had anticipated the occurrence and/or magnitude of the upcoming force stimulus, they would have made errors in maintaining a static posture against the load. This rarely happened, as participants failed to meet our arm 3D position criteria in ~2% of trials (see Methods). Finally, we believe that if participants had tried to anticipate the occurrence and/or magnitude of the force stimulus, this anticipation would have likely weakened towards the end of the trial while participants experienced the actual external force. Lastly, previous^42^ and current findings indicate that participants that exhibited similar PSE did not share similar synergistic patterns (Fig. S5 in Supplementary material), thus ruling out the possibility that the measured synergies arose from a common strategy of predicting the force stimulus.

In the present paper, we defined the parameter *w* of equation (5) in a different way relative to our previous work^42^ by considering the regression coefficient relating individual muscular activity to the external force. To test whether this different way of defining *w* might have caused a poorer description of perception by the MCM (Fig. 4), we used again the previous method on the current data. We found that the two MCM R^2^ distributions, obtained by the previous and current methods of computing *w*, were not significantly different (for details see S1.4 and Table S3 in Supplementary Material).

### Open questions and directions for future research

The current study suggests that perceptual decision about force may depend on the lower-level modulation of activity of a subset of muscles and higher-level translation of these sensorimotor signals into decision variables. While the present work cannot provide direct evidence of these perceptual decision mechanisms, an objective for future studies will be to correlate the temporal evolution of perceptual decision making, motor modules, and motor performance. Additionally, brain imaging would provide further insights into high-level control of perception and action in these perceptual tasks, as done in a recent study of tactile discrimination^43^.

Moreover, future studies should explore active sensing strategies through different dynamic scenarios to better understand the interplay between prior knowledge about the sensory stimulus and movements aimed at accumulating information. For example, participants could combine prior belief about stimulus presentation with patterns of movements aimed to reduce uncertainty of the information collected through movement (information sampling^44^). A recent study^45^ has shown that an active sensor algorithm based on Bayes optimal strategy allowed estimation of the contribution of prior knowledge of stimulus statistics and the amount of information provided by eye movement during a categorization task.

In summary, we found evidence of the potential role of muscle activation patterns in active perception of force. Thus, our findings point to a new research avenue that goes beyond the investigation of muscle synergies in the motor domain, and explores the potential advantage of a modular organization of motor control for active sensing and perception.

## Methods

### Participants

Ten right-handed participants (5 females, mean age = 25.4 ± 5.5 years; mean height = 1.69 ± 6.3 m; mean weight = 65.1 ± 8.8 Kg) participated in the study. All participants were naïve with respect to the aim of the experiment and gave their written informed consent. None of them had neuromuscular disorders and all had normal or corrected-to-normal vision. All the experimental procedures have been approved by the Ethical Review Board of the Fondazione Santa Lucia and have been conducted according to the Declaration of Helsinki.

### Apparatus

The experimental setup has been described in detail in a previous report^42^. Briefly, different magnitudes of upward forces (*F*) were applied on participants’ forearm by means of a load (counterweight, *Cw*) set on the balancer of a passive device (i.e., Track-Hold, TH; Fig. 1a). Point of force application (i.e., device attachment on the arm) was adjusted for each participant along their forearm to produce a torque acting on both shoulder and elbow joints^42,46^. Upward forces ranged from 0 to 30 N (resolution: 0.5 N). Visual feedback of target position and participants’ forearm movements was rendered as two 3D spheres (7.5 and 5.5 cm diameters, respectively) on a screen in front of the participant (gray and black circles, respectively; Fig. 1a). Vision of the arm was occluded throughout the experiment by a screen. Before the experiment, participants aligned their upper arm with the trunk (0° shoulder flexion and adduction) and their forearm parallel to ground (90° elbow flexion). To avoid the TH device from touching participants’ trunk, they were asked to position the flex the upper (~15° shoulder flexion) and outward (~15° shoulder adduction) with respect to the trunk (Fig. 1a). This initial arm posture was recorded before the first trial and used as a reference arm posture throughout the entire experiment. Each deviation greater than 5° from the initial arm posture was cued to the participant by changing the color of the cursor, and participants were asked to return to the original upper limb configuration. Average arm postures during force application were 13.4° ± 1.6°, 16.4° ± 3.8° and 88.1° ± 1.6° for shoulder flexion, shoulder adduction and elbow flexion, respectively. The present setup differs from those used by previous studies investigating isometric force production, where participants were constrained to produce/resist forces along a given plane with gravity compensation^26,41,47,48^. In our task, participants had to resist a force while maintaining a constant arm configuration over four degrees of freedom (elbow flexion, shoulder flexion and abduction, humeral external rotation) in a quasi-isometric condition.

### Procedures

On each trial, participants were asked (1) to judge the presence of an upward force applied on their right forearm while maintaining a quasi-isometric arm posture, and (2) to answer the question: “*Do you perceive an upward force applied on your arm?”*. At the beginning of each trial, participant’s arm rested on a tripod located in the same vertical and sagittal plane of the target area, but approximately 5 cm to the right (Fig. 1a). Once the *Cw* was placed on the TH balancer, to start each trial participants were asked to make a smooth and slow leftward movement to bring the arm position sphere inside the target sphere. Once the arm reached the target position, subjects had to meet two criteria before being asked to report the presence/absence of the upward force applied on their forearm (yes/no answer): they were required to (1) remain in the target arm position for at least 2 seconds, and (2) position their arm such that both target and arm position spheres overlapped within ± 2 cm, while keeping the shoulder and elbow angles within 5.0° from the angles associated with initial posture. There was no time constraint for providing an answer. Participants entered their responses by pressing one of two keys on a response box held in participants’ left hand. Positive answers denoted a detection of an upward force acting on the arm, whereas negative answers denoted perception of no external force. Once participants provided an answer, they were asked to return to the resting position.

The order of force presentations followed a double interleaved UP-DOWN^49^ staircase method^24^, where stimulus level on each trial was selected with respect to participant’s answer provided in the preceding trial (Fig. 1b). Upward force was increased after a negative answer and decreased after a positive one. This method allows reaching a force detection threshold that produced 50% of positive answers (i.e., Point of Subjective Equality, PSE; Fig. 1b). Importantly, this adaptive method provides a force presentation sequence that accounts for across-trials and within-subjects variability in detecting the presence of the force in each participant. The experiment ended after 13 inversions of the participant’s answers. The total duration of the experiment was approximately 1 hour.

### Data acquisition and EMG preprocessing

Arm posture was recorded using an electro-magnetic motion tracking system (Fast-Track Mid-Range 3D Guidance Trackstar, Ascension, Footscray, Australia) and the TH device. The Fast-Track tracked the position of wrist (styloid process of the ulna), elbow (epicondylus lateralis) and shoulder (acromion) joints by means of three active markers (sampling frequency: 80 Hz). The TH device (100 Hz sampling rate) was used to store the point of force application and update the visual feedback position. Both FastTrack and TH position data were low-pass filtered at 10 Hz with a second order zero phase-shift Butterworth filter and linearly extrapolated from 80 Hz (FastTrack), and from 100 Hz (TH) to 1000 Hz to match the sampling rate of electromyography (EMG) recordings. Surface EMG was recorded throughout each trial with active bipolar surface electrodes (DE 2.1; Delsys, Boston, MA) from eight muscles (Fig. 1a inset): brachio-radialis (BrRad); biceps brachii, long head (Bic); triceps brachii, long head (Tric); trapezius upper (TrapU); trapezius middle (TrapM); latissimus dorsi (LatD); deltoid, anterior (DeltA) and deltoid, posterior (DeltP). These muscles were selected to cover a range of arm muscles that were mostly involved in the quasi-isometric force produced to resist the TH upward torque. We selected a group of muscle consisting of muscles that were anatomically separated (i.e., attached to either elbow or shoulder joints) and functionally different (e.g., shoulder flexion or elbow extension). EMG signals were band-pass filtered (20–400 Hz) and amplified (total gain 1000, Bagnoli-16, Delsys Inc.). The signal was then digitized at 1 KHz (PCI-6035E, National Instruments, Austin, TX). EMG for each trial were digitally full-wave rectified and low-pass filtered by a 2^nd^ order zero-phase shift Butterworth at 25 Hz for smoothing. After visual inspections of position and EMG data, we excluded from further analysis those trials where muscles waveform showed artifacts and/or the aforementioned position criteria were not satisfied. The average percentage of excluded trials across participants was 2.4 ± 2.1% of all trials. For the remaining trials, we analyzed EMG during the last 30% of the trial duration (Fig. 1a inset) as this epoch was temporally close to participants’ answer. EMG dataset was processed differently according to the model considered. For the most contributive model (MCM), we calculated the mean absolute values (MAV) of EMG activity for each muscle. MAV values were then normalized (i.e., normMAV) with respect to the maximum MAV recorded from that muscle across all upward forces presented (see *The most contributive model* below). For the muscle synergy model (MSM), we identified the muscle synergies or modules characterizing participants’ muscle activity involved in the isometric task.

### Muscle synergy extraction

Muscle synergies were extracted using nonnegative matrix factorization^50^ (Matlab *nnmf* function). This decomposition method assumes that the net muscle activation pattern vector **m** is composed by a linear combination of few muscle synergies ***W***_i_, each of which is scaled by the synergy activation coefficient *c*_**i**_:

1 $${\bf{m}}={\sum }_{n=1}^{N}{c}_{i}\cdot {{\boldsymbol{W}}}_{{\boldsymbol{i}}}$$

where each value of ***W***_i_ represents a muscle with a specific contribution (unit magnitude) to the whole muscle synergy *i* (Fig. 2a, left plot). Muscle synergies ***W*** are assumed to be constant across trials, namely describing the spatial pattern of muscle activity of each synergy^24^. Coefficients *c*_i_ characterize the way the entire muscle synergy is modulated in time. We examined the change in the activation of each synergy, i.e., increase of coefficient values as a function of the upward force (i.e., Fig. 2a, middle plots). Specifically, we modeled the relation between the upward forces and the modulation of the synergy activation coefficient to classify each muscle synergy as either related to the upward forces (i.e., *force-related synergies*) or as weakly influenced by the external force (Fig. 2a, right plot). For each participant, we iterated the extraction of ***W***_i_ and *c*_i_ by varying the number (N*syn*) of muscle synergies (*i* from 1 to 8), where each vector *c*_i_ contains the associated synergy activation coefficient at sampled time point of each trial. Thus, at each iteration, ***W*** was an 8 x N*syn* matrix and *c* was a N*syn* x time (last 30% of duration of each trial) matrix. We then averaged each *c*_i_ in time to obtain a single average activation coefficient value per trial, namely obtaining a *c* matrix N*syn* x *k* (number of trials).

Before the synergy extraction procedure, we performed a unit-variance normalization to ensure that muscle activity from each muscle was equally weighted^48^. Reconstruction of observed EMG activity of individual muscles was obtained through a weighted sum of the muscle synergy vectors. The significance of pattern decomposition was assessed using the percentage of variance accounted for i.e., VAF, extracted by comparing the reconstructed muscle activity to the observed activity for each muscle (local VAF) and the entire (global VAF) EMG data set^51^. VAF was defined as 100·uncentered Pearson correlation coefficient, whose advantage is to assess both shape and magnitude of actual and reconstructed curves^41^. The minimum number of synergies required to achieve both a local VAF > 75% and global VAF > 90% was defined as the minimum activity pattern that best accounts for the EMG data variance^48^. We refer to this group of synergies as *task-related synergies*. When the global criterion was satisfied, but local reconstruction of one muscle was lower than 75%, an additional synergy was considered. The new synergy was added to the original group of synergies only if adding it increased the global VAF more than 3%. Synergy extraction procedure was repeated 200 times to obtain mean and confidence intervals (95^th^ percentile) of VAF distributions. Distribution of the global VAF was compared to the distribution of VAF values obtained from structure-less (random) data^27^ by means of non-parametric Wilcoxon signed rank test (i.e., right sided test of median). As in^27^, random data were obtained by independently reshuffling the observed sample across time for each muscle and then filtering (10 Hz cutoff) the obtained random sample.

### Psychometric and muscle-synergy curve

We modeled both perceptual detection and muscle synergy modulation as function of the force stimuli. Specifically, we investigated whether and to what extent the modulation of muscle synergy activations can account for participants’ probability of detecting an external force. For each participant, we analyzed the relationship between the reported force detection and applied upward force by fitting a Generalized Linear Model^52^ (probit link function, MERpsychophysics package, *R*):

2 $${Y}_{t}^{\ast }={\beta }_{0}+{\beta }_{1}{F}_{t}$$

where *F*_t_ is the upward force at trial *t*, *β*_0_ and *β*_1_ are the intercept and slope of the linear regression, respectively, and ${Y}_{t}^{\ast }$ is a latent response variable linked to the response variable *Y*_t_ at trial *t* (i.e., *‘yes/no’*), through the probit link function (${\Phi }^{-1}$, *psychometric curve*). On the other hand, we described the modulation of each muscle synergy through two possible outcomes (*Y*) at each trial *t*, i.e., synergy activation coefficient higher or lower than mean activation (dashed line in Fig. 2a, middle plot). This description was obtained by examining the across-trials distribution of the activation coefficients *c* of each muscle synergy *i*. For each synergy *i*, in each trial, we defined an ‘increase’ in synergy activity when *c*_i_ exceeded the average activation of that synergy, i.e., *p*(*c*_*it*_> ${\bar{c}}_{i}$); *Y*_*it*_ = 1, ‘decrease’ otherwise, i.e., *Y*_*it*_ = 0 (dots above or below dashed lines, respectively, in Fig. 2a, middle plots). We then calculated the across-trials probability of each external force to elicit an activation increase of each muscle synergy (dots in Fig. 2a, right plots). The relation between the modulation of each muscle synergy activation and the upward force applied was quantified by fitting a Generalized Linear Mixed Model^52^ (GLMM, glmer function, *R*). This analysis extended the Probit analysis to clustered categorical data by considering the overall synergy activation variability into the fixed effect predictor -i.e., vector of upward forces *F*- and random effects predictors, i.e., differences in muscle synergies modulation for each synergy and across synergies:

3 $${Y}_{ik}^{\ast }={\beta }_{0}+{\beta }_{1}{F}_{k}+{\varepsilon }_{ik}+{\mu }_{i}$$

where *β*_0_, *β*_1_ are the intercept and slope of the linear regression, respectively, *F*_*k*_ is the force at trial *k*, and ${Y}_{ik}^{\ast }$ is the latent variable linked (${{\rm{\Phi }}}^{-1}$) to the response variable *Y*_*ik*_ -i.e., ‘increase/decrease’ in activation- for the muscle synergy *i* at trial *k*. Finally, *ε*_*ik*_ and *μ*_*i*_ represent the variability taken into account within and between muscle synergy, respectively. Importantly, GLMM analysis predicts the probabilities of activation increase/decrease of each synergy (individual muscle synergy curve, Fig. 2b) and the overall synergies activation (MSM curve; Fig. 2c, black curve) as function of upward force. From the individual synergy analysis, the slope of the final model captures the extent to which each synergy is modulated as a function of force stimuli curve. The inclusion of the slope as random parameter of the model was supported by the chi-square statistics indicating a significant increase in model fitting. Statistical significance of output model coefficients (*β*_0_, *β*_1_) was assessed by Wald test^52^. Goodness of fit of both psychometric and muscle synergy curve was assessed separately through deviance test. Furthermore, we tested that the deviance resulting from fitting the full model having force as predictor was statistically lower than the deviance of the intercept-only model where force was set to zero. Finally, to account for synergies that either increased or decreased their activation with respect to a change of upward force (i.e., negative or positive slope in Fig. 2a, right plot), we used the absolute value of the slope. This allowed us to focus on how synergy modulation related to the final perception about force regardless of its biomechanical contribution in our task, e.g., flexor versus extensor muscle activity. Thus, unless otherwise specified, we will hereafter refer to both types of synergy modulation as probabilities of activation increase.

### Force-related synergy identification and MSM curve

The overarching hypothesis of our study is that the modulation of a subset of muscle synergies or modules recruited in an isometric force task can account for a substantial portion of participants’ perceptual variance in detecting force stimuli, i.e., Muscle Synergy Model (MSM) hypothesis. To test this hypothesis, we identified the subset of muscle synergies engaged to counteract the external force and whose modulation statistically related to the force stimuli, i.e., individual muscle synergy curves with statistically significant slope (*force-related synergies*; green and red curves in Fig. 2b). We identified the smallest group of force-related synergies through an iterative procedure, identifying and excluding at each iteration the synergy modules not significantly related to the force stimuli. GLMM analysis identified probabilities of activation increase of each muscle synergy recruited during the task (${Y}_{ik}^{\ast }$ eq. 3). From the models obtained by GLMM analysis, we defined N*syn* nested models, each consisting of a reduced number of muscle synergies (*i* in eq. 3). At each iteration, the muscle synergy that was less related with the force stimuli (Wald statistics on curve parameters) was left out (blue curve in Fig. 2b). Subsequently, we obtained the overall GLMM probability of activation increase for each probabilistic nested model, each composed of different numbers of muscle synergies. The nested model with minimum Δ Akaike Information Criterion (AIC), calculated by subtracting the lowest AIC value from all others nested models’ AIC values, was considered the smallest subset, within the task-related synergy group, accounting for external force changes (MSM curve). The iteration procedure stopped when one of the following conditions was met: 1) only one model had a ΔAIC lower than 10, hence suggesting that the nested model considered is as informative as the best model with higher numbers of parameters (see S1.2, Supplementary Material); or 2) the model with lowest ΔAIC was composed of only two muscle synergies and no more nested models could be extracted (see example in Fig. 2b,c). The goodness of fit of the MSM curve obtained by the iteration was tested by means of the deviance ratio test^52^ (see below). The reliability of the MSM curve to describe the relation between the modulation of the synergy subset and the external force changes was tested through Wald statistics on curve parameters.

### The most contributive model

While the MSM hypothesis posits that muscle synergies involved in motor task account for participants force detection, the most contributive model approach, i.e., MCM, assumes that force detection relies on a linear combination of EMG from individual muscles scaled by their modulation with respect to the force stimuli (S1.3, Supplementary Material). According to this model, for each force stimulus the probability of force detection (p(‘yes’)) can be described by a weighted sum of the normalized mean absolute EMG activities of all muscles recorded during the task, *PoolEMG*_*i*_:

4 $$PoolEM{G}_{i}={\sum }_{k=1}^{8}(normMA{V}_{k}\cdot {w}_{k})$$

Where *normMAV*_*k*_ is calculated as described above (see *Data acquisition and EMG preprocessing*), *i* is the trial number, *k* is a muscle, and *w* is the multiple regression coefficient specifying the direction and contribution of each muscle to the overall muscular activity associated to the external upward force. From the across-trial distribution of the PoolEMG, we obtained a curve describing the across-trial probability of each force magnitude to elicit a PoolEMG greater than its mean^42^ (MCM curve). As done for MSM, we identified the best MCM curve as the nested model showing the best trade-off between simplicity (i.e., number of muscles) and similarity to the psychometric curve (pseudo-R²). The best MCM was extracted from a group of 7 nested models leaving the least contributive muscle out at each iteration (iterative multiple regression, stepwise elimination procedures^42^). This procedure allowed to identify the number of most contributive muscles composing the simplest MCM that were most likely to account for a high percentage of perceptual answer variance, i.e., pseudo-*R*² ≥ 0.60, this value being the highest perceptual variance explained by the model reported in^42^. Use of the regression coefficients to define *w*, which differs from our previous approach^42^, was motivated by the assumption that it better describes the muscle activity modulation across trials, rather than considering only the smallest and largest force stimuli. By comparing the MCM *R*^²^ values associated with the two methods of calculating *w*, the use of regression coefficients provided a higher across-subjects MCM *R²* (see Table S3, Supplementary material).

### Comparison between model curves and perceptual performance

We assessed which of the two models, MSM and MCM, better described perceptual behavior by means of three different criteria: deviance ratio, *Δ* parameters (slope, *Δ*_*slp*_, and PSE, *Δpse*), and Efron pseudo-R².

We first fitted each model on the probability of force detection (*DEVmuscular* and *DEVperceptual*, respectively). We computed the deviance ratio *γ* as follows:

5 $$\gamma =\frac{DE{V}_{muscular}}{DE{V}_{perceptual}}$$

where *γ* is distributed as χ² with 2 degrees of freedom, *DEV*_*muscular*_ is the deviance obtained by fitting either MCM or MSM curves on the probabilities of detections, and *DEV*_*perceptual*_ is the deviance obtained by fitting the psychometric curve on the same probabilities. Therefore, by testing *γ* to be lower than the criterion value, we tested that the psychometric curve and a given model were not significantly different in describing participants’ perceptual behavior^53^.

Similarity between model and perceptual functions was further quantified by subtracting the parameters characterizing a given model curve from the perceptual curve, i.e.,*Δ*_*pse*_ and *Δ*_*slp*_. These parameters denote the accuracy and precision of detection, respectively. The distributions of the Δ parameters (obtained through bootstrapping 1000 sampling for each participant) were submitted to a one-sample Wilcoxon signed rank test. This was used to test the null hypothesis that their medians were not significantly different from zero, thereby indicating a non-significant difference between a given model and perceptual functions.

Efron’s pseudo-R² was obtained as follows:^54^

6 $${R}^{2}=1-\frac{\,{\sum }_{i=1}^{N}{({P}_{i\phi }-{P}_{i\omega })}^{2}}{{\sum }_{i=1}^{N}{({P}_{i\phi }-\overline{{P}_{\phi }})}^{2}}$$

Where *P*_*φ*_ and *P*_ω_ are the probabilities of a ‘yes’ answer and the probability of overall synergy activation increase, respectively, for MSM and the probability $PoolEM{G}_{i} > \overline{PooEMG}$ for MCM on the *i*^*th*^ trial. Importantly, Efron’s pseudo-R² quantified the extent to which the MSM and MCM models could account for perceptual variance while detecting the external force. Subsequently we tested what model accounted for more than 60% of perceptual variance, this being the largest variance of perceptual performance accounted for by muscle-metric curves^42^. Finally, we cross-validated the MSM hypothesis by comparing perception to a muscle synergy curve derived from 8 muscle synergies (i.e., full set of synergies) and another synergy curve composed of the muscle modules that had been excluded by the ΔAIC procedure, i.e., non-perceptual synergies whose modulation was weakly or not related to the force stimuli.

### Muscle model composition and synergy contribution

We calculate the coefficient of shared muscles to quantify the similarity in the muscle composition of MCM and MSM. Specifically, this coefficient represents the number of muscles that were composed both the best MCM and characterized the MSM muscle modules with a high activation strength (> 0.5). Coefficients of shared muscles lower than 0.5 indicate that MSM accounts for perceptual variance by means of different muscles than those in the MCM. Conversely, if the MSM represented the same most contributive muscles as MCM, the coefficient would be equal to 1. We also tested whether a better description of perceptual behavior provided by the MSM is due to the greater contribution of force-related synergies in reconstructing the measured EMG dataset. To this end, we compared the ability of force-related synergies against the non-perceptual synergies subset to reconstruct EMG measured from each muscle.

### Software

Motion capture analysis, EMG preprocessing, synergy extraction procedure and all the related statistics were carried out through both built-in and customized functions written in *Matlab R2014a* (Mathworks, Natik, MA). GLM, GLMM and probabilistic model comparisons procedure were performed by means of both built-in and customized functions written in *R3.3.1* and based on lme4.0^52^ package. We estimated model and psychometric curve parameters through the Bootstrap Method included in the MERpsychophysics *R-packages* (see http://mixedpsychophysics.wordpress.com).

## Supplementary information

Supplementary information

## Data Availability

Please contact S.T. to obtain a copy of the data and/or the custom Matlab and R codes.
