# Supplementary material for: Motor modules account for active perception of force
Source: Sci Rep. 2019 Jun 20;9:8983. doi: 10.1038/s41598-019-45480-w (PMC6586614; doi:10.1038/s41598-019-45480-w)
Supplement: Supplementary file 1 — Supplementary information [file 41598_2019_45480_MOESM1_ESM.docx]

**Motor modules account for active**

**perception of force**

Simone Toma,^1,2^ Marco Santello^2^

*^1^Laboratory of Neuromotor Physiology, Santa Lucia Foundation, Rome, Italy 00179*

*^2^School of Biological and Health Systems Engineering, Arizona State University, Tempe, AZ 85287-9709*

*Short Title*: Muscle synergies account for active perception of force

Corresponding author: Simone Toma, School of Biological and Health Systems Engineering, 501 East Tyler Mall, ECG Building, Suite 334, Arizona State University, Tempe, AZ 85287-9709, simone.toma@asu.edu

**Supplementary Material**

S1.1 Synergy Extraction. The identification of the minimum number of task-related synergies through local and global criteria ensured that both each muscle and overall muscle activity could be reconstructed with a good level of accuracy. In some cases (e.g., subjects 9 and 10, Fig. S2), the increase of variance accounted for (VAF) values with respect to the number of synergies does not have the usual ‘knee-shape’ trend often reported in literature^1,2^. For these participants, adding more synergies did not significantly enhance the global VAF, this being always over 90%. In contrast, adding more synergies for these subjects improved single muscle activity reconstruction, thereby satisfying the local criterion. We interpret this result as evidence that our EMG reconstruction accounted for relevant changes of individual muscle activity, rather than random variations in the data. We note that, in the present task, participants had to resist a force while maintaining arm configuration, against gravity, over four degrees of freedom across shoulder and elbow in a quasi-isometric condition. One can assume that inter-subject differences in agonist-antagonist muscle activity elicited by the requirement of maintaining a static posture influenced the total number of task-related synergies across participants. This interpretation is supported by data shown in Fig. 3 showing a higher variability of the number of task-related synergies (3 to 7) than force-related synergies (2 to 3). We note that this comparison effectively removes the influence of the postural maintenance requirement on muscle synergies.

1.2 Force-related synergy iteration procedure. The interpretation of the level of information provided by each model was based on Burnham and Anderson heuristic table suggesting that ΔAIC higher than 10 represents a nested model significantly less informative than the best model^3^. Occasionally, during the iteration procedure the GLMM optimization routine either failed to converge, or the nested model with lowest ΔAIC exhibited a slope coefficient not statistically significant. In these two cases, the nested model with lowest ΔAIC was discarded and the model with the second lowest ΔAIC was considered as MSM for further analysis.

1.3 MCM framework. The rationale underlying the MCM framework is based on studies investigating the sense of force during tasks involving single or pairs of functionally-related muscles, i.e., inserting on the same joint. This previous work suggested that the perception of force and weight is relative, rather than absolute^4^, namely it results from the relation between descending commands and the characteristics of individual muscles activated during performance of the task, e.g., fatigability or changes in muscle biomechanics^5-7^, density of corticospinal projection^8,9^, maximal force-generating capacity of the muscle^5,10,11^, the group of muscles involved in the motor task^8^, muscles’ anatomical location and number of motor units innervating those muscles^12,13^. The MCM relies on the assumption that the muscles that are recruited the most during force production are also the muscles that best encode changes in the external force, hence influencing perception. This framework is also supported by empirical evidence suggesting that activation of multiple muscles during force production aims to minimize the overall signal-dependent noise^13^ through flexible muscle recruitment based on individual mechanical action^14^.

1.4 Effect of *W* calculation on MCM *R²* values. In agreement with our previous work^15^, in the present study we found that the MCM is characterized by a R^2^ distribution whose median is not significantly different than 0.6 (Fig. 7 in ^15^ and Fig. 4b, black empty bar). Furthermore, when we re-calculated *w* and MCM curves using the present data by means of the same method used in ^15^ - i.e., *w* obtained from the difference between normMAV at 30 N and 0 N force stimuli - we did not find consistent reduction of the MCM associated with the regression coefficient (Table S3). Importantly, Wilcoxon two-sided paired samples test performed on the overall participants R^2^ distributions revealed no significant difference (*p* = 0.23) between the two methods of calculating *w*.

**Supplementary figures**

**
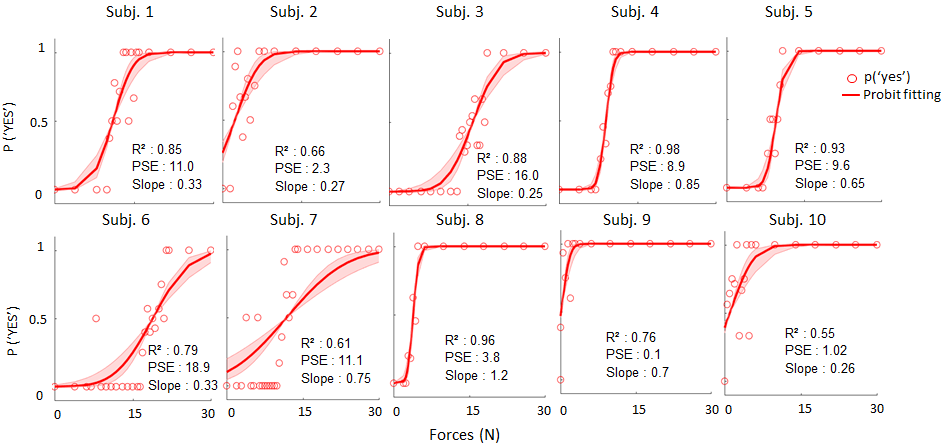
**

**Fig. S1. Psychometric curves.** Individual probabilities of detection (circles) and psychometric curves (probit fitting) describing subjects’ probability of ‘yes’ answers as a function of force stimuli. Shaded area represents 95% confidence intervals of fit parameters obtained through bootstrap.

**
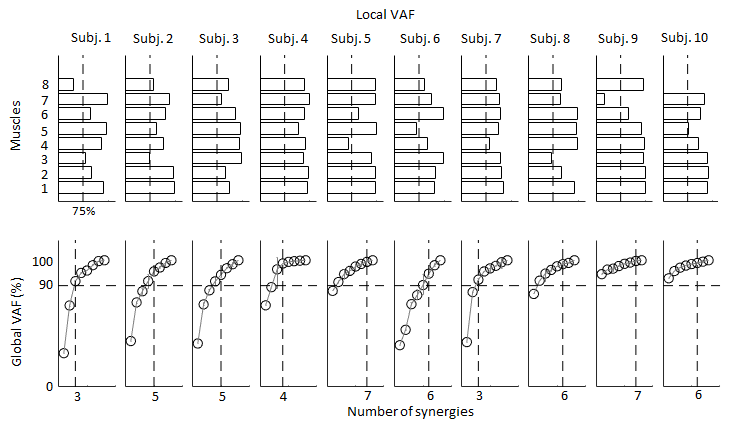
**

**Fig. S2. Synergy Extraction.** *Upper plots:* Local variance accounted for (VAF) for each subject and muscle, i.e., 1) brachioradialis; 2) biceps brachii; 3) triceps brachii; 4) trapezius middle; 5) trapezius upper; 6) latissimus dorsi; 7) deltoid anterior; 8) deltoid posterior. Vertical dashed lines stand for the local criteria, i.e. VAF = 75% (see *muscle synergy extraction* in Methods, main text). *Lower plots:* Global VAF values (lower plot) for each subject as function of the number of muscle synergies. Horizontal dashed line in the lower plot indicates the global criteria, i.e., VAF = 90%, used to select the least number of muscle synergies to reconstruct the whole EMG dataset, i.e., task-related synergies. Vertical dashed lines in both plots indicate the number of task-related synergies for each subject.


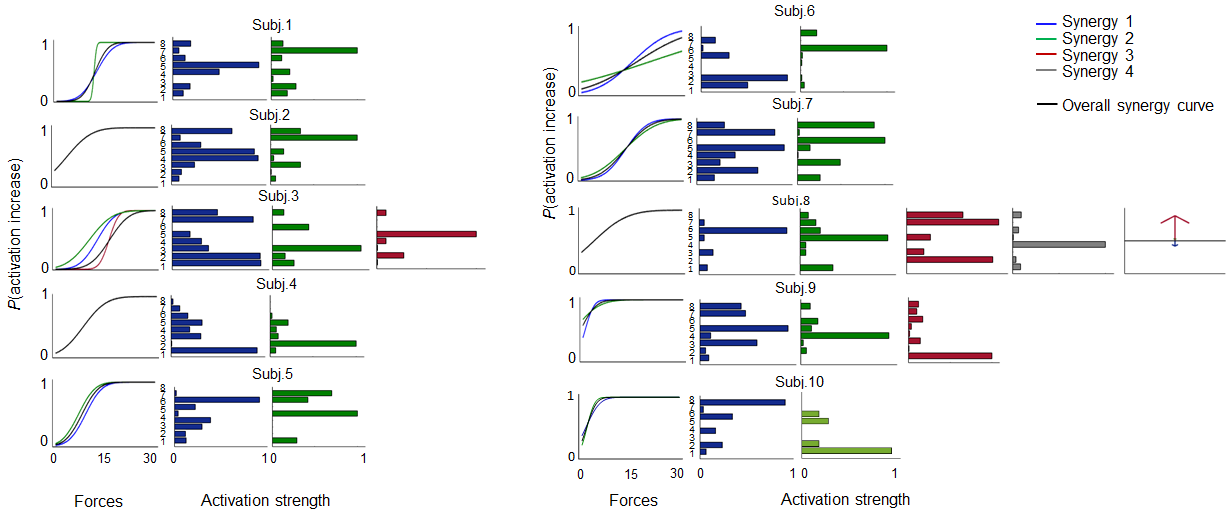


**Fig. S3. Force-related synergies.** Each curve represents GLMM predictions of activation increase of each individual synergies (colored) and the overall (black) synergy subset. Bars represent activation strength of each muscle composing each one of the force-related synergy. Muscles numbering in bars plot: 1) brachioradialis; 2) biceps brachii; 3) triceps brachii; 4) trapezius middle; 5) trapezius upper; 6) latissimus dorsi; 7) deltoid anterior; 8) deltoid posterior.


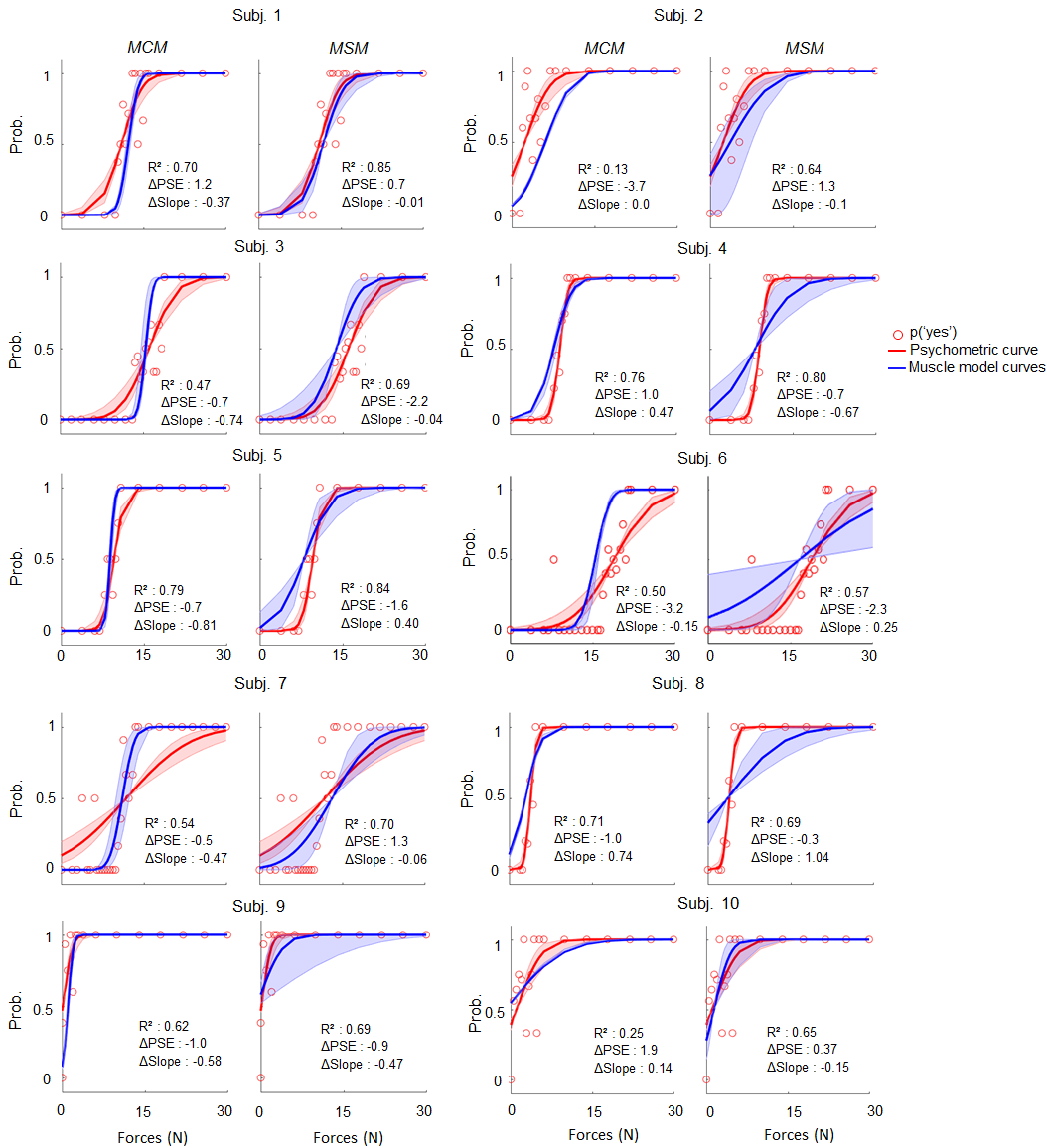


**Fig. S4. Perceptual, MCM and MSM curves.** Each plot depicts individual probability of answer yes (red dots), the psychometric curve (red) and either the MCM (blue, left) or the MSM (blue, right) curve. In each plot, shaded area represents 95% confidence intervals of the slope and intercept distributions obtained by bootstrap. Efron pseudo-R² values represent the amount of perceptual variance that either the MCM or the MSM curve accounts for. ΔPSE and ΔSlope were obtained by subtracting muscle curve parameters from the perceptual one (see *Comparison between muscle model curves and perceptual performance*, Methods section).


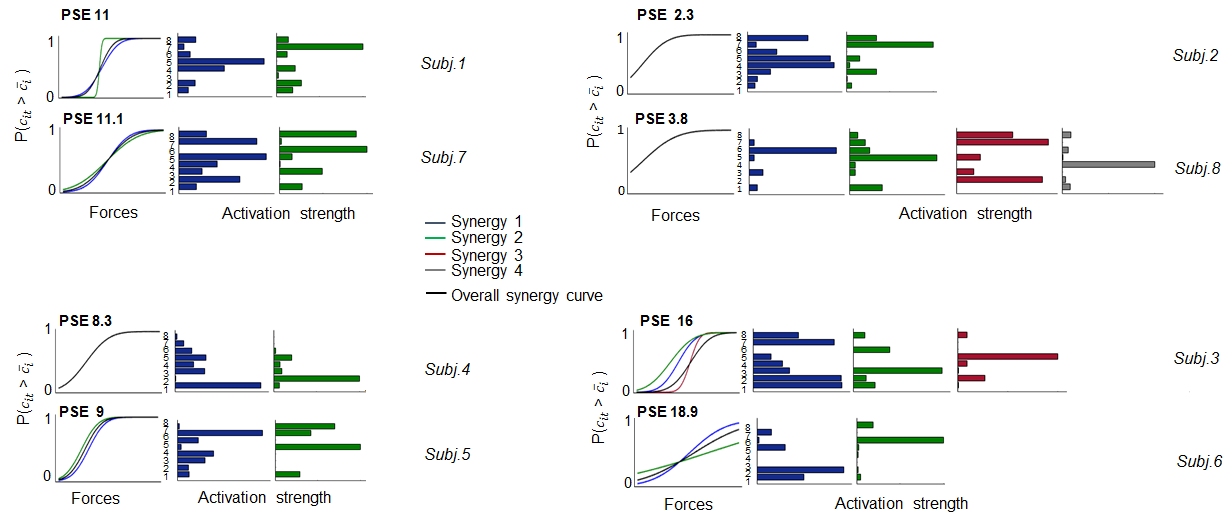


**Fig. S5. Comparison between participants with similar PSE.** For each subject, each curve represents GLMM predictions of activation increase of each individual synergy (colored) and the overall (black) synergy subset. Bars represent activation strength of each muscle composing each one of the force-related synergy. Muscle numbering in bars plot: 1) brachioradialis; 2) biceps brachii; 3) triceps brachii; 4) trapezius middle; 5) trapezius upper; 6) latissimus dorsi; 7) deltoid anterior; 8) deltoid posterior. Note that participants exhibiting similar perceptual thresholds (PSE) do not share similar pattern of muscle co-activation, i.e., different muscle activation strength (bars).

**
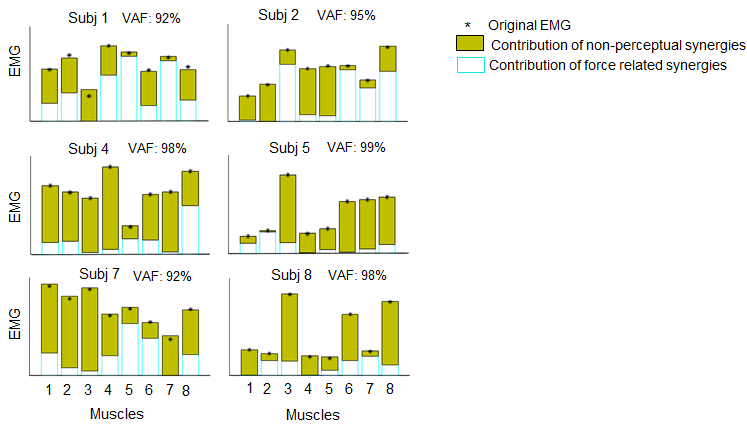
**

**Fig. S6. Contribution of synergy subsets to EMG dataset reconstruction.** Perceptual (cyan empty bars) and non-perceptual (filled green bars) synergy subset contribution to reconstruction of original EMG activity (VAF) of each muscle calculated for those 6 participants not shown in the main text (Fig. 6). Asterisks denote original EMG of each muscle. VAF values refer to the global variance accounted for by the task-related muscle synergy, namely when perceptual and non-perceptual synergies subsets were merged for EMG reconstruction. Muscles numbering is the same as in Fig. S5.

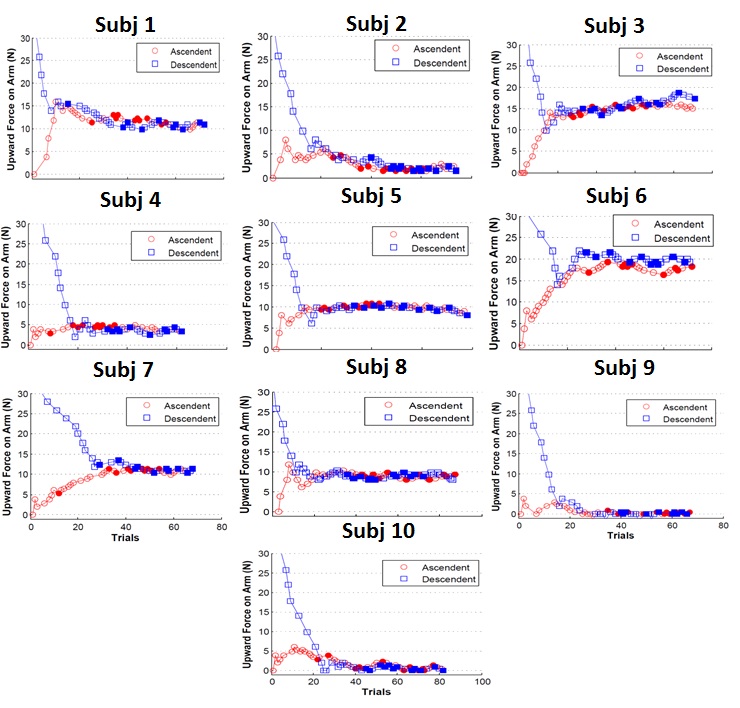


**Fig. S7. Individual double staircase force stimuli presentation.** Blue squares and red circles represent the trend of descendent and ascendant force stimuli presentations, respectively, across trials. At each trial, force stimuli intensity was set based on subjects previous answer, i.e., no = increase in force intensity; yes = decrease in force intensity). As it can be noted the difference in force stimuli presented by the two staircases is very high at the beginning of the experiment but it decreases when the two presentation sequences converge. All our participants exhibited trends of staircase presentations that converged on a relatively stable threshold value, hence suggesting that participants used a consistent strategy to judge force stimuli throughout the experiment.

**Tables**

|  | *subj 1* | *subj 2* | *subj 3* | *subj 4* | *subj 5* | *subj 6* | *subj 7* | *subj 8* | *subj 9* | *Subj 10* |
| --- | --- | --- | --- | --- | --- | --- | --- | --- | --- | --- |
| Slope | 0.33 ± 0.1 | 0.27 ± 0.1 | 0.25 ± 0.1 | 0.85 ± 0.4 | 0.65 ± 0.2 | 0.33 ± 0.0 | 0.75 ± 0.0 | 1.2 ± 0.3 | 0.7 ± 0.2 | 0.26 ± 0.2 |
| PSE | 11.0 ± 0.6 | 2.3 ± 0.6 | 16.0± 0.7 | 8.9 ± 0.1 | 9.6 ± 0.2 | 18.9 ± 0.7 | 11.1 ± 2.0 | 3.8 ± 0.1 | 0.1 ± 0.1 | 1.02 ± 0.1 |
| Wald Statistics | 2.85** | 2.36* | 2.60** | 3.37** | 2.9** | 3.01** | 2.46* | 3.50** | 2.91** | 2.52* |
| Deviance test (χ²) | 15.6(17) | 21.2(16) | 10.7(22) | 21.2(17) | 5.8(16) | 13.1(25) | 31.3(26) | 5.9(15) | 11.0(16) | 24.8(19) |

**Table S1. Psychometric curve parameters.** Estimated ± SE intercept and slope values of each subject’s psychometric curve. Statistical significance of curve parameters was assessed by Wald statistics (z-score). Goodness of fit analysis was assessed by testing that the deviance between the Probit fitting and the observed probabilities were not significantly different than zero (χ², 95%). Values in parentheses are the number of points used for the probit fitting and deviance test calculation.

|  | ${MCM}_{1}$ | ${MCM}_{2}$ | ${MCM}_{3}$ | ${MCM}_{4}$ | ${MCM}_{5}$ | ${MCM}_{6}$ | ${MCM}_{7}$ | ${MCM}_{8}$ |
| --- | --- | --- | --- | --- | --- | --- | --- | --- |
| N of muscles | 8 | 7 | 6 | 5 | 4 | 3 | 2 | 1 |
| R² | 0.55 ± 0.07 | 0.55 ± 0.07 | 0.55 ± 0.06 | 0.56 ± 0.07 | 0.56 ± 0.08 | 0.58 ± 0.07 | 0.53 ± 0.06 | 0.46 ± 0.18 |
| *p-value* | 0.62 | 0.62 | 0.62 | 0.62 | 0.62 | 0.84 | 0.77 | 0.16 |

**Table S2. MCM nested models.** Each column shows the number of muscles composing the nested model (first row), the median ± SE of the amount of perceptual variance accounted for (Efron pseudo-R², middle row), and the p-value (lower row) from a two-sided Wilcoxon signed rank statistics testing Efron pseudo-R² median to be different than 0.6.

|  | *Subj 1* | *Subj 2* | *Subj 3* | *Subj 4* | *Subj 5* | *Subj 6* | *Subj 7* | *Subj 8* | *Subj 9* | *Subj 10* | *Overall* |
| --- | --- | --- | --- | --- | --- | --- | --- | --- | --- | --- | --- |
| *R²* $W_{diff}$  (Toma et al.,2016) | 0.65 ± 0.15 | 0.06 ± 0.4 | **0.47 ± 0.3**** | **0.77** ± 0.1** | **0.80 ± 0.1**** | 0.42 ± 0.3 | **0.55 ± 0.2**** | 0.48 ± 0.2 | 0.56 ± 0.2 | **0.27 ± 0.1**** | 0.51 ± 0.2 |
| *R²* $W_{regrCoeff}$  (Regression coefficient) | **0.70 ± 0.1**** | **0.13 ± 0.4**** | 0.47 ± 0.3 | 0.76 ± 0.1 | 0.79 ± 0.1 | **0.50 ± 0.2**** | 0.54 ± 0.2 | **0.71 ± 0.1** | **0.62 ± 0.2**** | 0.25 ± 0.1 | 0.58 ± 0.2 |
| *z-*value | -19.85 | -10.87 | 3.22 | 2.89 | 5.64 | -13.57 | 5.67 | -25.43 | -13.34 | 6.24 | *p = 0.23* |

**Table S3. Effect of *W* calculation on MCM *R²*** **values.** Median ± SE of MCM curve Efron pseudo-R² values (i.e., amount of perceptual variance described by MCM) associated to*w* calculated either as in Toma et al, 2016 (*R²* $W_{diff}$, upper row) or obtained from the regression coefficients (*R²* $W_{regrCoeff}$, middle row). Statistics is reported as z-value obtained from a paired two-sided Wilcoxon signed rank test for each participant’s curves. Positive z-values indicate *R²* $W_{diff}$ to be significantly greater than *R²* $W_{regrCoeff}$ , and vice versa for negative values. Values in bold denote the distribution with higher median value. Although the difference between the two methods produces significant changes in the individual MCM *R²,* the effect is not consistent across subjects (half of the sample exhibited *R²* $W_{diff}$ > *R²* $W_{regrCoeff}$ and the other half vice-versa). Importantly, the use of regression coefficients to estimate *W* resulted in a more significant increase of *R²* $W_{regrCoeff}$ values with respect to *R²* $W_{diff}$ , i.e., overall negative z-values are more than twice the overall positive z-values. The last column shows across-subjects median ± SE and associated statistics. Overall, the test suggests that the two methods of extracting *W* does not significantly influence MCM R².

**References**

1. d’Avella, A., Portone, A., Fernandez, L., Lacquaniti, F. Control of fast-reaching movements by muscle synergy combinations. *J Neurosci*. **26**, https://doi.org/10.1523/JNEUROSCI.0830-06.2006 (2006).
2. Roh, J., Rymer, W.Z., Beer, R.F. Robustness of muscle synergies underlying three-dimensional force generation at the hand in healthy humans. *J Neurophysiol* **107**, 2123-42, https://doi.org/10.1152/jn.00173.2011 (2012).
3. Lewandowsky, S., Farrel, S. Computational modeling in cognition: principles and practice*. Sage*, California (2011).
4. Jones, L.A. Perceptual constancy and the perceived magnitude of muscle forces. *Exp Brain Res* **151**, 197-203, https://doi.org/[10.1007/s00221-003-1434-4](https://doi.org/10.1007/s00221-003-1434-4) (2003).
5. Carson, R.G., Riek, S. & Shahbazpour, N. Central and peripheral mediation of human force sensation following eccentric or concentric contractions. *J Physiol* **539**, 913-25, https://doi.org/10.1113/jphysiol.2001.013385 (2002).
6. Cafarelli, E., Bigland-Ritchie, B. Sensation of static force in muscles of different length. *Exp. Neurol*. **65**, 511-523, https://doi.org/10.1016/0014-4886(79)90040-2 (1979).
7. Monjo, F., Forestier N. Muscle spindle thixotropy affects force perception through afferent-induced facilitation of the motor pathways as revealed by the Kohnstamm effect. *Exp Brain Res* **236**, 1193-1204, https://doi.org/[10.1007/s00221-018-5207-5](https://doi.org/10.1007/s00221-018-5207-5) (2018).
8. Gandevia, S.C., Rothwell, J.C. Knowledge of motor commands and the recruitment of human motoneurons. *Brain*. **110**, 1117-1130 (1987).
9. Gandevia, S.C., Kilbreath, S.L. Accuracy of weight estimation for weights lifted by proximal and distal muscles of the human upper limb. *J. Physiol.* **423**, 299-310 (1990).
10. Monree, H.M., Klein C., Marcora S.M. Perception of effort reflects central motor command during movement execution. *Psychophysiology*.**49**, 1242-53, https://doi.org/10.1111/j.1469-8986.2012.01399.x (2012).
11. Gandevia, S.C. The sensation of effort co-varies with reflex effects on the motoneuron pool: evidence and implications. *Int. J. Ind. Erg*. **13**, 41-49, https://doi.org/10.1016/0169-8141(94)90063-9 (1994).
12. Hamilton, A.F.dC., Jones, K.E., Wolpert, D.M. The scaling of motor noise with muscle strength and motor unit number in humans. *Exp. Brain Res.* **157**, 417-430, https://doi.org/[10.1007/s00221-004-1856-7](https://doi.org/10.1007/s00221-004-1856-7) (2004).
13. Jones, K.E., Hamilton, A.F.dC., Wolpert, D.M. Sources of signal dependent noise during isometric force production. *J Neurophysiol*. **88**, 1533–1544, https://doi.org/10.1152/jn.2002.88.3.1533 (2002).
14. Kutch, j.j., Kuo D.K., Block A.M., Rymer, W.Z. Endpoint force fluctuations reveal flexible rather than synergistic pattern of muscle cooperation. *J Neurophysiol*. **100**, 2455–71, https://doi.org/10.1152/jn.90274.2008 (2008).
15. Toma, S., Lacquaniti F. Mapping muscle activation to force perception during unloading. *PLoS One* **11**, https://doi.org/10.1371/journal.pone.0152552 (2016)
